# Supplementary material for: Therapy and Outcome of Prolonged Veno-Venous ECMO Therapy of Critically Ill ARDS Patients
Source: J Clin Med. 2023 Mar 25;12(7):2499. doi: 10.3390/jcm12072499 (PMC10094941; doi:10.3390/jcm12072499)
Supplement: Supplementary file 1 [file jcm-12-02499-s001.zip › Supplementary Materials.pdf]

Supplement S1: Selected therapy parameters of the treatment duration groups.

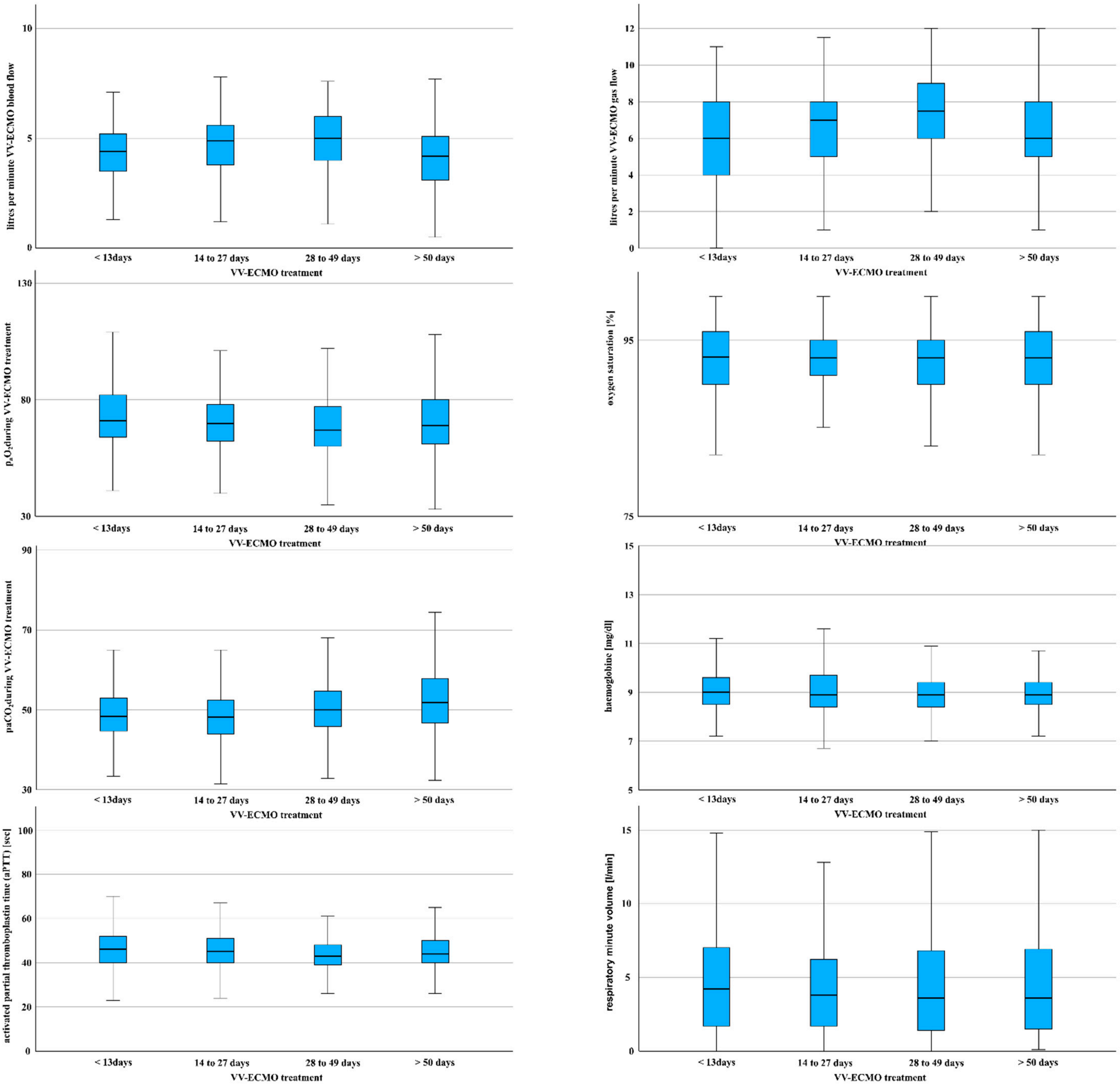

Box and whisker plots of the daily results of selected laboratory, ventilator and treatment parameters, subdivided according to the duration of VV-ECMO therapy. Abbreviations: dl, deciliters; l, litre; min minutes; ml; p_aCO_2, arterial carbon dioxide partial pressure; p_aO_2, arterial oxygen partial pressure; sec, seconds; VV-ECMO, veno-venous Extracorporeal Membrane Oxygenation

Supplement S2: Breathing rate and tidal volume over time separated by survival and grouped by treatment duration.

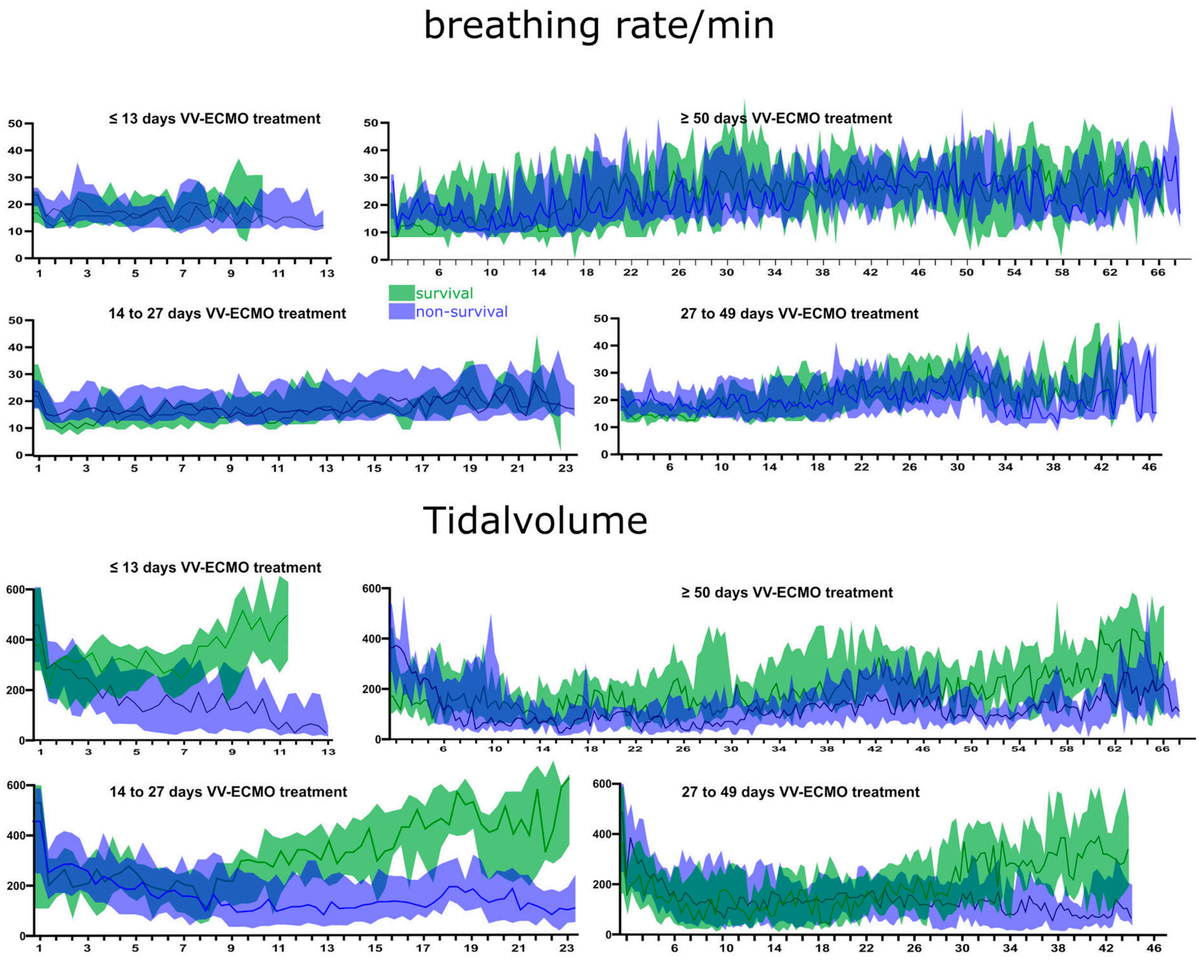

Graphical visualisation of breathing rate and tidal volume over the course of the treatment days as median including the corresponding interquantile range as coloured area.
